# Wintering Habitat Model for the North Atlantic Right Whale (*Eubalaena glacialis*) in the Southeastern United States

**DOI:** 10.1371/journal.pone.0095126

**Published:** 2014-04-16

**Authors:** Timothy A. Gowan, Joel G. Ortega-Ortiz

**Affiliations:** Florida Fish and Wildlife Conservation Commission, Fish and Wildlife Research Institute, St. Petersburg, Florida, United States of America; Pacific Northwest National Laboratory, United States of America

## Abstract

The coastal waters off the southeastern United States (SEUS) are a primary wintering ground for the endangered North Atlantic right whale (*Eubalaena glacialis*), used by calving females along with other adult and juvenile whales. Management actions implemented in this area for the recovery of the right whale population rely on accurate habitat characterization and the ability to predict whale distribution over time. We developed a temporally dynamic habitat model to predict wintering right whale distribution in the SEUS using a generalized additive model framework and aerial survey data from 2003/2004 through 2012/2013. We built upon previous habitat models for right whales in the SEUS and include data from new aerial surveys that extend the spatial coverage of the analysis, particularly in the northern portion of this wintering ground. We summarized whale sightings, survey effort corrected for probability of whale detection, and environmental data at a semimonthly resolution. Consistent with previous studies, sea surface temperature (SST), water depth, and survey year were significant predictors of right whale relative abundance. Additionally, distance to shore, distance to the 22°C SST isotherm, and an interaction between time of year and latitude (to account for the latitudinal migration of whales) were also selected in the analysis presented here. Predictions from the model revealed that the location of preferred habitat differs within and between years in correspondence with variation in environmental conditions. Although cow-calf pairs were rarely sighted in the company of other whales, there was minimal evidence that the preferred habitat of cow-calf pairs was different than that of whale groups without calves at the scale of this study. The results of this updated habitat model can be used to inform management decisions for a migratory species in a dynamic oceanic environment.

## Introduction

The North Atlantic right whale (*Eubalaena glacialis*) is highly endangered, and although it has received protection from the Endangered Species Act of 1973 and the Marine Mammal Protection Act of 1972, the species remains well below its optimum sustainable population level [1]. Threats to the recovery of this species include entanglement in fishing gear and collisions with ships [2]. Effective management decisions, and assessing risk of injury and mortality from these threats, require knowledge of right whale habitat preferences to identify areas where whales are likely to occur.

**Figure 1:**
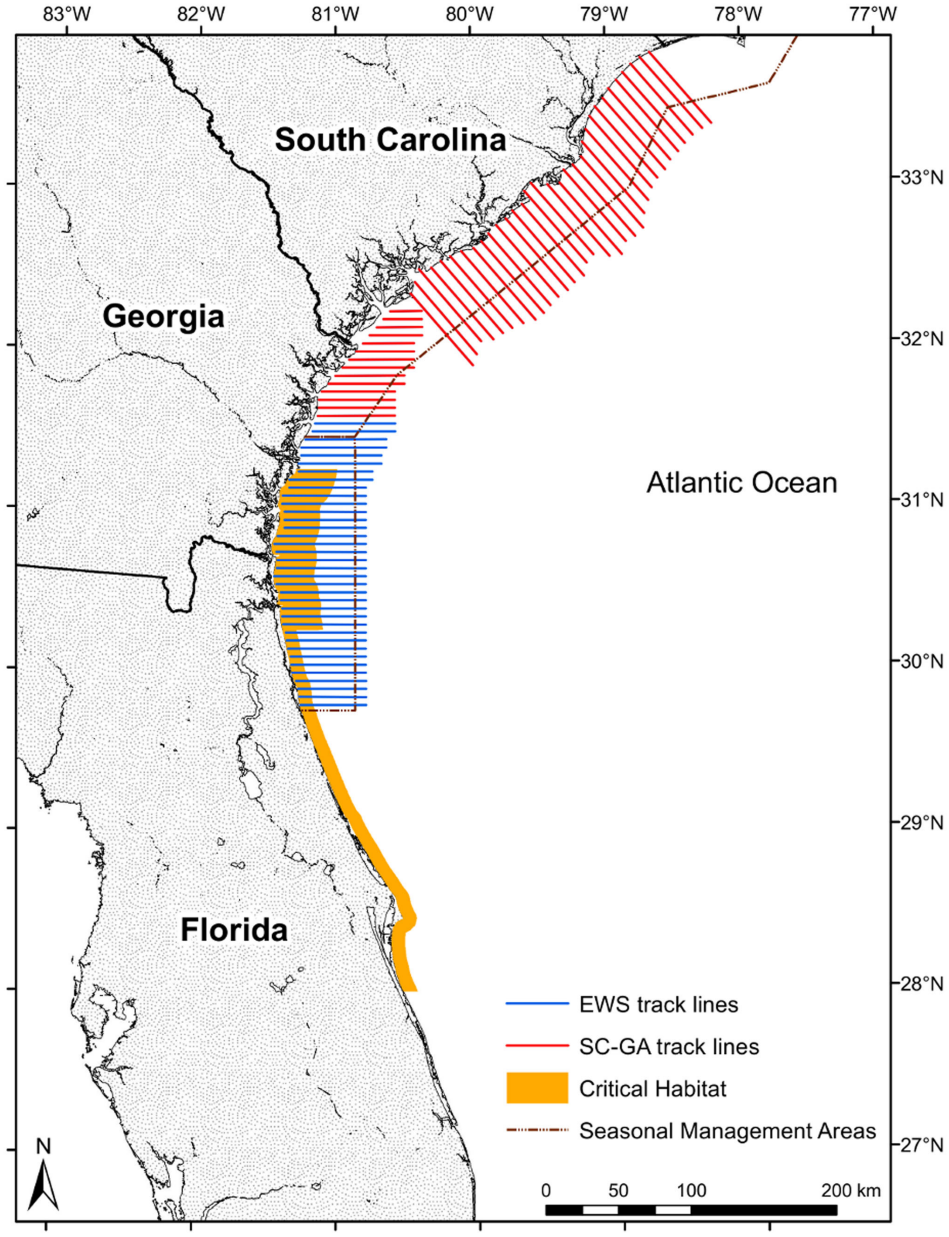
Aerial survey track lines for right whales in the southeastern United States. Transect lines are from the Early Warning System (EWS) and South Carolina/Georgia (SC-GA) survey areas. Critical habitat and seasonal management area boundaries are included for reference.

Western North Atlantic right whales occupy nearshore habitats of North America, with their primary feeding grounds from spring through autumn off the coast of New England and eastern Canada. The coastal waters off the southeastern United States (SEUS), however, have been identified as their primary calving grounds, with cow-calf pairs and some juveniles and adults occupying the waters off Florida (FL) and Georgia (GA) during winter months (December–March) [3]. Several management measures have been established in the SEUS due to threats from human activities in this region, especially the risk of ship strikes from the high volume of shipping traffic associated with the ports of Jacksonville, FL, Fernandina, FL, Brunswick, GA, and Savannah, GA. Measures designed to reduce the likelihood of ship collisions with right whales in high-risk areas include a mandatory ship reporting system, seasonal management areas with ship speed restrictions, recommended transit lanes for large ships, and aerial surveys during the calving season (Figure 1). In an effort to enhance protection measures for North Atlantic right whales, aerial surveys in the SEUS have been supported since the early 1990s by a collection of agencies, including the National Oceanic and Atmospheric Administration National Marine Fisheries Service (NOAA Fisheries), the US Coast Guard, the US Navy, and the US Army Corps of Engineers. As part of that effort, a multiagency, coordinated survey network, termed the Early Warning System (EWS), has been in place since the 1993/1994 calving season [4]. The level of effort varied in the early surveys, but a core survey area off the Florida-Georgia border was regularly flown [5]. In 2003 EWS survey lines were modified as depicted in Figure 1 to provide consistent coverage of nearshore waters from Butler Beach, FL (29.76° N) to Sapelo Sound, GA (31.56° N). A primary objective of these surveys is to locate right whales in the area and relay this information to mariners; other objectives include monitoring population vital rates and human-related injuries with the aid of photo identification and characterizing whale habitat use and distribution. Additional surveys off South Carolina and northern Georgia (SC-GA) were implemented in the 2004/2005 calving season (Figure 1) with similar objectives, providing coverage from Sapelo Sound, GA (31.56° N) to North Myrtle Beach, SC (33.82° N).

Effective management actions rely on the ability to know where and when right whales are likely to occur, yet the boundaries of management areas are typically static in time and space, while whale distribution varies spatially and temporally in response to dynamic environmental conditions [6], [7]. Additionally, right whale distribution changes seasonally as whales migrate between wintering grounds in the SEUS and feeding grounds at higher latitudes. If whale distribution can be modeled and predicted from environmental variables and behavioral patterns, management strategies can be developed that take into account system variability and uncertainty. Several predictive habitat models have been developed for the right whale wintering grounds in the SEUS [5], [8], [9], [10]. However, these models did not analyze the most current survey data, including surveys with more consistent protocols (see Methods) and data from the recent SC-GA surveys, which have significantly expanded coverage in the northern section of the wintering ground. Accurate inferences from species distribution models are frequently limited to the extent (spatial, temporal and environmental) of the input data (*e.g.*, [11]), and development of these models, particularly for rare and endangered species, should be an iterative process validated and informed by newly available data [12], [13]. Right whale abundance (sighting rates) observed during more recent surveys in the northern portion of the wintering ground was not consistent with predictions from previous models. Additionally, none of the previous right whale habitat models for the SEUS accounted for latitudinal migration patterns or for differences in the probability of whale detection due to variable platforms (type of plane) and survey conditions, as has been done in other studies (*e.g.*, [14]).

In this study, we characterized right whale habitat in the SEUS using generalized additive models (GAMs) to relate whale sightings from aerial surveys to static and dynamic environmental variables. We modeled the occurrence (presence-absence) and relative abundance of whales using a hurdle model. We analyzed recent survey data (from 2003/2004 through 2012/2013), with spatial coverage ranging from southern Florida through South Carolina. Our model was developed at a two-week temporal resolution, and it included survey effort, adjusted for survey platform and sea state, and inter- and intra-annual effects as covariates. We generated predictions from this model, and the results demonstrate that right whale distribution varies within and among years. Additionally, we compared environmental conditions for sightings with a calf present to those without calves to determine whether cow-calf pairs utilize habitat that is different from other whales that migrate to the SEUS.

## Methods

### Ethics Statement

Whale surveys were conducted under permits #0594-1467, #594-1759-00 and #15488 issued to the Georgia Department of Natural Resources and permits #655-1652 and #14233 issued to Scott Kraus (New England Aquarium) by NOAA Fisheries. All research protocols were reviewed by NOAA’s Office of Protected Resources and complied with the Endangered Species Act and the Marine Mammal Protection Act.

### Aerial Surveys

Aerial surveys for right whales have been conducted in the SEUS during the right whale calving season (December–March) since 1991, but effort intensity and spatial coverage have varied considerably among years outside of a core EWS area [5]. We analyzed survey data from 2003/2004 through 2012/2013 for this study because survey methods were standardized and the location of EWS transect lines was consistent during these seasons. Sightings and effort data for these surveys were provided by the North Atlantic Right Whale Consortium [15]. Since 2003/2004, EWS surveys have been flown along fixed track lines running east-west, spaced 3 nautical miles (5.56 km) apart, and extending 24–34 nautical miles (44–63 km) offshore (Figure 1). Surveys were conducted from a Cessna 337 Skymaster or a de Havilland DHC-6 Twin Otter aircraft with a target altitude of 1000 ft (305 m). EWS surveys were designed to be flown daily during the calving season and complete the defined survey track lines, dependent upon weather conditions and aircraft availability. The SC-GA surveys began in 2004/2005 using Skymaster aircraft and the same data-collection protocols as in EWS surveys but with dedicated track lines running east-west and spaced 3 nautical miles (5.56 km) apart in the southern portion of the survey area and running northwest-southeast and spaced approximately 4 nautical miles (7.52 km) apart in the northern portion (Figure 1). Additional coastal (“Florida nearshore,” *sensu* [5]) surveys south of these track lines were flown in some years. Most effort during coastal surveys was within 10 nautical miles (18.5 km) off and parallel to the shoreline (generally in a north-south direction).

During aerial surveys, one observer on each side of the aircraft searched for right whales, and a computer program was used to automatically record geographic location (latitude, longitude, and altitude) obtained from the aircraft’s GPS every 10–30 seconds. Environmental conditions (sea state, visibility, weather) were recorded at the beginning of the survey and updated as conditions changed. When a right whale was observed, the aircraft deviated from the designated track line to approach the sighting location. Whale locations were recorded as the GPS location when the aircraft flew directly over the whale. After recording the behavior and number of whales and obtaining photographic documentation, the aircraft returned to the designated track line to resume survey.

### Survey Data Processing

Data recorded during each flight from all EWS, SC-GA, and coastal surveys were entered into a GIS (ArcGIS version 10.0, Esri Inc., Redlands, CA), and the equidistant Universal Transverse Mercator (UTM) projection was used for data analysis. We filtered survey data to only include portions considered “on-effort”: sea state ≤3 (Beaufort scale), altitude ≤365 m, visibility ≥3.7 km, and flying along a designated track line (*i.e.,* not in transit or circling a whale). Whale sightings were considered on-effort if that was the survey status at the time of the initial sighting. We removed all verification survey sightings and their associated effort (*i.e.*, surveys conducted to locate and verify a reported whale sighting) and all duplicate sightings (*i.e.*, whales already sighted on the same survey, as verified by photo identification). Each segment of a flight path was buffered on both sides with an effective search width, according to survey platform and sea state, to estimate the searched area. To determine effective search widths, perpendicular sighting distances were calculated from survey track lines and right whale sighting locations [16], and the multiple-covariate distance sampling (MCDS) engine in Distance 6.0 software was used to model detection probabilities [17]. Separate detection functions were created for each survey platform (Skymaster and Twin Otter aircrafts) using sea state as a covariate in MCDS, resulting in distinct effective search widths (range = 1.3–2.2 km) for each platform/sea state combination. Because the Skymaster has flat windows resulting in a blind spot beneath the aircraft, a section corresponding to 0.186 km (unpublished field data) on both sides of the flight path was removed from the estimated searched area for surveys from this platform. The Twin Otter had no blind spot because it has bubble windows that allowed observers to search directly under the aircraft.

**Figure 2:**
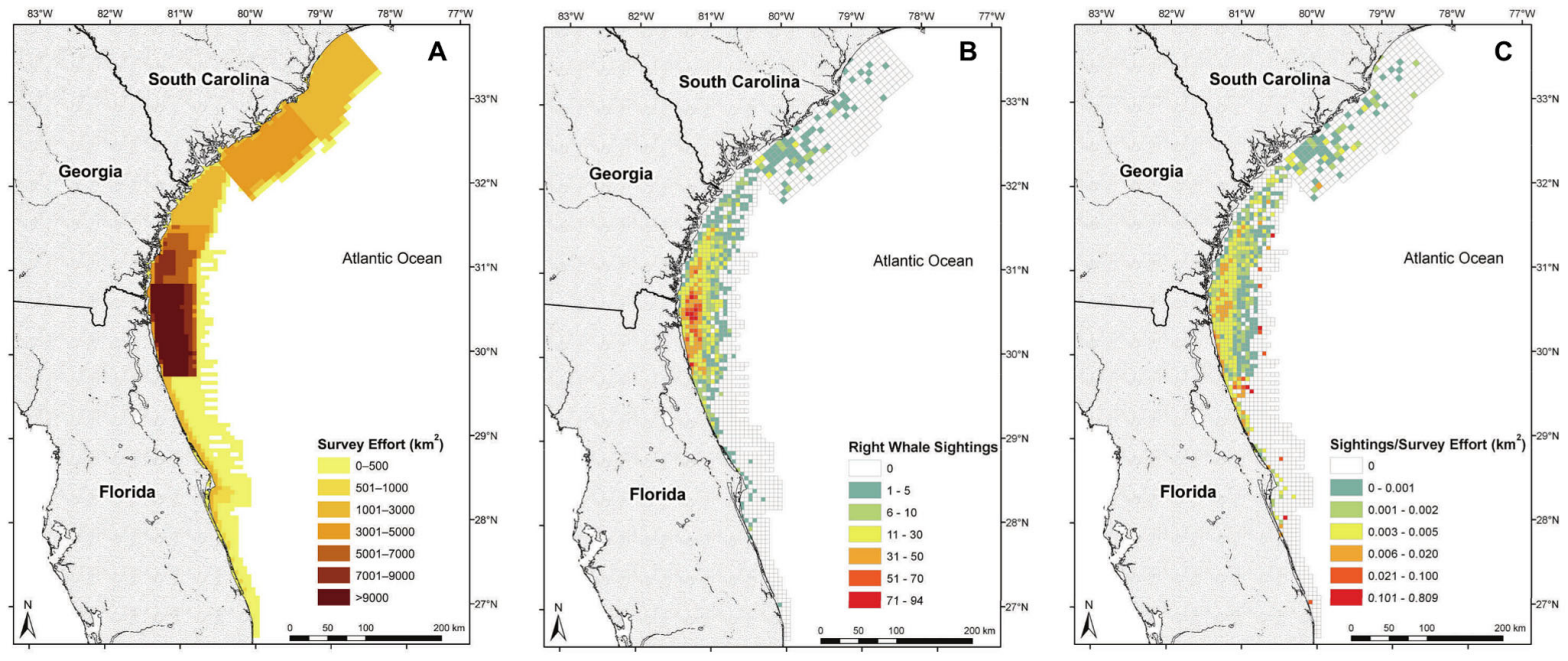
Aerial survey effort and right whale sightings in the southeastern United States. Values represent cumulative area surveyed (A), cumulative number of whales sighted (B), and number of whales sighted divided by area surveyed (C) per grid cell between December 2003 and March 2013 while observers were on-effort.

A composite sampling grid for the study area was constructed to accommodate disparities in track line spacing and orientation, consisting of 5.56−×5.56-km cells oriented east-west in the south and 7.52−×7.52-km cells oriented northwest-southeast in the north (Figure 2). We overlaid the sampling grid onto the study area so the track lines bisected the grid cells, allowing all survey effort within a cell to be associated with a single track line. Because whale sightings were so rare, we aggregated all survey and environmental data into the grid cells at a semimonthly (approximately 2 week) temporal resolution–either the 1st through the 15th day of a month (A) or the 16th through the last day of a month (B)–to increase the number of records with whale presence, while maintaining a time frame with relatively stable environmental conditions. Thus each survey year consisted of eight semimonthly periods (December A through March B). Each cell summarized over a semimonth is hereafter considered a sampling unit. For each semimonth, the searched area (km^2^) from all surveys during that period was summed within each grid cell and used as the measure of survey effort. Two sampling units (survey effort >340 km^2^) were identified as outliers on a Cleveland dotplot [18] and were excluded from analysis.

### Environmental Data

Geographic locations (UTM easting and northing) were taken at the center point of each grid cell. Distance to shore was calculated as the distance between the center point of each cell and the high-resolution NOAA composite shoreline GIS layer (http://shoreline.noaa.gov). Bottom depth data were obtained from the 30-arc-second-resolution data set of the General Bathymetric Chart of the Oceans (http://www.gebco.net). Depth was calculated as the mean bottom depth of all values within each grid cell; cells with mean depth above sea level or deeper than 70 m (22 sampling units considered outliers) were excluded from analysis. Bottom slope (degrees) was derived from bathymetric data using Spatial Analyst in ArcGIS and was also summarized as the mean of values within each cell. Sea surface temperature (SST) was derived from Advanced Very High Resolution Radiometer (AVHRR) 1.47-km-resolution imagery from NOAA’s CoastWatch data set (http://cwcaribbean.aoml.noaa.gov/data.html) for the East Coast South region. Within each semimonth all available daily images with minimal cloud cover were downloaded (mean = 8.1 images), image pixels with cloud interference were removed, and the semimonthly mean SST was calculated at each pixel. SST for a sampling unit was calculated as the mean of all semimonthly mean SST 1.47 km pixels within each cell. The potential environmental predictors mentioned above were chosen based on previous studies of right whales in the SEUS [5], [8], [9], [10]. Additionally, the semimonthly mean SST data were used to estimate the location of the 22°C SST isotherm (Spatial Analyst in ArcGIS 10.0); this isotherm was chosen because 22°C is at the upper SST range for right whales in this region [5] and can be used as a proxy for the Gulf Stream boundary in winter [19]. Distance from this isotherm was calculated from the center point of each grid cell; distances for sampling units with SST >22°C were set as negative values, indicating that a cell was east or south of the isotherm.

### Model Framework

GAMs were used to relate the number of right whale sightings to possible predictor variables. GAMs are extensions of generalized linear models (GLMs) that allow for smooth, nonlinear functions of predictor variables determined by observed data rather than by strict parametric relationships [20], [21]. Like GLMs, GAMs use a specified error distribution for the response variable and a link function to relate the response variable to the predictor variables. Because the response variable, number of sighted whales, was overdispersed and zero-inflated due to the large number of sampling units (96%) with no sightings, we used a hurdle model [22]. A hurdle model consists of two steps: modeling presence-absence with a binomial distribution, and then modeling positive abundance, conditional on presence. Hurdle models are therefore useful for modeling zero-inflated data and allow the modeled process which determines presence to differ from the process which determines abundance [22]. We first used a quasibinomial distribution (to deal with excessive number of zeros) with a logit link to model presence-absence from all data. We then used a gamma distribution with a log link to model the number of whales from sampling units with whale sightings [23]. Predicted relative abundance can be calculated by multiplying the probability of occurrence, derived from the first model, by the expected number of whales, derived from the second model. Often, models of count data from wildlife surveys include effort as an offset term, assuming this variable as a linear predictor with a coefficient equal to one [21]; our data, however, did not meet this assumption (see Results), and we included effort as a smoothed covariate. GAMs were constructed using the R statistical software (version 3.0.1) with the mgcv package (version 1.7-24; [24]). The mgcv package determines the appropriate degrees of freedom (df) and smoothing for each predictor variable by minimizing the generalized cross validation (GCV) score [21]. To avoid overfitting by the model and to limit complexities of the smoothing function to an ecologically interpretable relationship, we set the basis dimension parameter to 3, thereby limiting the maximum df for each term to 2 (*e.g.*, [25], [26]).

The response variable for the hurdle model was the total number of right whales sighted in each sampling unit. An individual whale could have been sighted multiple times within the same semimonth, and we did not correct for availability bias (*e.g.*, whales submerged at time of survey); therefore, our model is representative of the relative spatial distribution of whales rather than absolute abundance or density. Possible predictor variables included depth, distance to shore, SST, distance to the 22°C isotherm, and survey effort. Survey year was included as a factor variable to account for inter-annual variation in the total number of right whales in the SEUS, and the interaction between northing and semimonthly period was used to account for intra-annual shifts in distribution resulting from the timing of the whale migration. Easting was ultimately excluded as a predictor variable in favor of more informative environmental variables with which it was correlated. Slope was also excluded because all survey effort occurred on the continental shelf within a narrow range of values (0.01–1.29°), and it was consistently found to be the least significant term in a stepwise selection (see Table S1). Although collinearity was present among some of our explanatory variables, its impact was minimized by removing easting and slope to reduce variance inflation factors for the remaining predictors to <4.8 [27], employing penalized regression splines with shrinkage in the mgcv package to estimate smoothing functions [28], [29], and limiting model predictions to the range of sampled data [29].

### Model Selection and Validation

Model selection was accomplished with a forward stepwise selection procedure, using the following evaluation criteria: model GCV scores, percentage of deviance explained, and analysis of deviance tests. Starting with a null model, each term was added individually; the term resulting in the lowest GCV score was included in the next step. At each step, analysis of deviance was used to determine whether increasing model complexity with the addition of the selected term significantly improved the model. A term was not included in the final selected model if the confidence interval for the fitted response included zero for all observed values of the term or if the model at the previous step (which did not contain the term) had a lower model GCV score [24].

A five-fold cross-validation was used to evaluate each candidate model’s performance in predicting novel data [30]. Model training was based on a random subset of 80% of the data and used to predict the validation subset (remaining 20%). Average squared prediction error (ASPE) was calculated from the predicted and observed number of whales sighted in the validation subset. This cross-validation was run five times, and the mean ASPE was calculated for each model to assist in model selection. Final specification of the selected best model used to estimate smoothing functions and create prediction maps was based on the complete data set.

Spatial autocorrelation in species distribution models can be problematic because it violates the assumption that residuals are independently distributed. We examined whether spatial autocorrelation was present by calculating Moran’s *I* of deviance residuals of predicted relative abundance for all 80 semimonths (8 semimonths/year × 10 years) using the R-package spdep (version 0.5-56; [31]). Moran’s *I* was calculated as a global statistic, considering all grid cells as neighbors, with an inverse distance weighting such that nearby grid cells exerted greater influence. Moran’s *I* ranges from −1 (perfect negative correlation) to +1 (perfect positive correlation), with values near 0 indicating no spatial autocorrelation present.

### Segregation of Calves

Studies of baleen whales, including southern right whales (*Eubalaena australis*), indicate that cows with calves may prefer environmental features different from those used by other demographic groups on wintering grounds [32], [33], and it has been suggested that cow-calf pairs receive fitness benefits by segregating themselves from the harassment of other whales [34] or by avoiding areas with more predators [8]. We therefore compared environmental variables (depth, distance to shore, SST, distance to the 22°C isotherm, and UTM northing) at locations of sightings with calves present to those without calves. For this analysis, we overlaid exact sighting locations on the original environmental GIS layers (not the sampling grid) to maximize resolution for each variable. SST values were estimated only for sightings that occurred on days for which SST images were available and at locations free of cloud interference. Isotherms were constructed as stated above, based on semimonthly mean SST data. We excluded all sightings that were not on-effort or those with an estimated depth above sea level due to data precision. Sightings were classified based on the presence of a calf, pooled across years, semimonths, and levels of survey effort, and compared using a Mann-Whitney test. Due to the potential effect of migration timing, we tested for differences in UTM northing in each semimonthly period using a series of pair-wise comparisons with sequential Bonferroni correction [35].

## Results

A total of 2191 surveys was flown in the EWS and SC-GA areas from December 2003 through March 2013, resulting in sightings of 3286 right whale groups and 7369 whales (not unique individuals, as some whales were resighted within and between seasons) while on-effort. The greatest concentration of both survey effort and sightings occurred in the EWS area near the coast of northern Florida and southern Georgia (Figure 2). A total of 56143 sampling units, containing 3104 sightings of 6953 whales, was retained for analysis after removing statistical outliers and those with missing SST values due to cloud interference.

### Occurrence Model

For the presence-absence model, the GAM stepwise selection procedure yielded higher explained deviances, lower GCV scores, and lower ASPE values at each step, signifying that models had a better fit as complexity increased (Table 1, Table S1A). This finding was supported by analysis of deviance tests at each step; the more complex models were consistently identified as the most parsimonious despite having more parameters (Table S2A). The selected best model included seven predictor variables as significant (survey effort, SST, distance to the shoreline, bottom depth, interaction between semimonthly period and UTM northing, survey year, and distance to the 22°C SST isotherm) and explained 22.8% of the total deviance (Table 1). This model had the lowest ASPE values for all validation data sets, demonstrating its superior ability to predict the spatial distribution of right whales from novel data in the study area.

**Table 1:** Summary of stepwise selection procedure for presence-absence model of right whales in the southeastern US.

Model	% Deviance	GCV	mean ASPE
null	0	0.3417	0.0392
s(SemiMonth:Northing)	10.3	0.3065	0.0377
s(SemiMonth:Northing)+s(DistToShore)	15.7	0.2882	0.0364
s(SemiMonth:Northing)+s(DistToShore)+Year	18.8	0.2777	0.0352
s(SemiMonth:Northing)+s(DistToShore)+Year+s(Effort)	20.9	0.2706	0.0345
s(SemiMonth:Northing)+s(DistToShore)+Year+s(Effort)+s(SST)	22.3	0.2658	0.0342
s(SemiMonth:Northing)+s(DistToShore)+Year+s(Effort)+s(SST)+s(Depth)	22.6	0.2647	0.0340
s(SemiMonth:Northing)+s(DistToShore)+Year+s(Effort)+s(SST)+s(Depth)+s(DistTo22Iso)	22.8	0.2642	0.0340

Predictor variables include interaction between semimonthly period and UTM northing, distance to the shoreline (DistToShore), survey year, survey effort, sea surface temperature (SST), bottom depth, and distance to the 22°C SST isotherm (DistTo22Iso). Smoothed covariates indentified by “s()”. Evaluation criteria include the proportion of deviance explained, generalized cross validation score (GCV), and mean average squared prediction error (ASPE) from a five-fold cross-validation.

**Figure 3:**
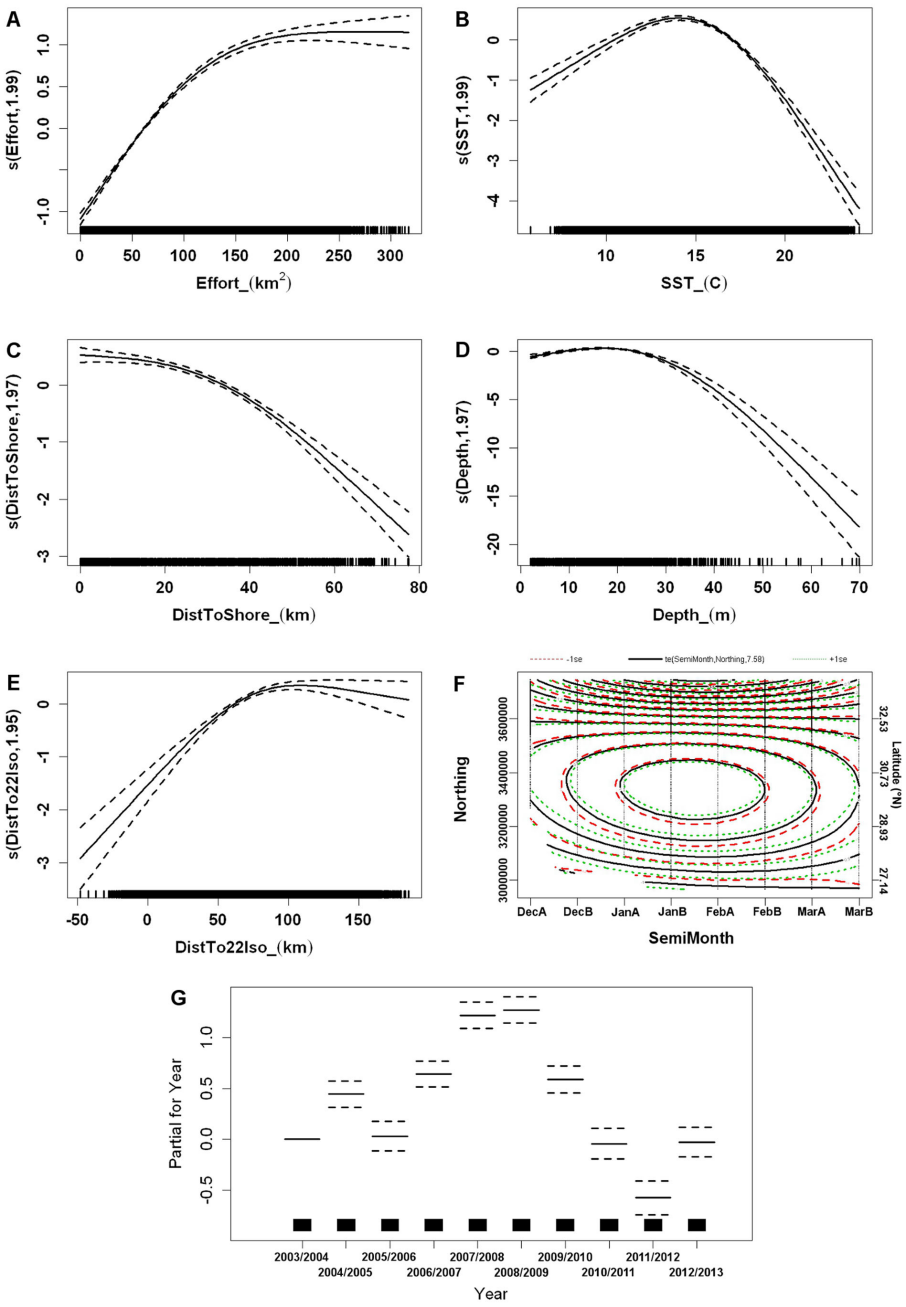
Smooth functions of predictor variables for presence-absence model of right whale sightings. Predictor variables include survey effort (A), sea surface temperature (B), distance to the shoreline (C), bottom depth (D), distance to the 22°C isotherm (E), interaction between semimonthly period and UTM northing (F), and partial effects for survey year (G). The y-axis is on the scale of the linear predictor, and dashed lines indicate ±2 standard errors. Estimated degrees of freedom for each smoothed variable are in parentheses on the y-axis; tick marks on the x-axis indicate all sampled values.

Smoothing functions for all terms in this model indicated nonlinear relationships and were consistent with the locations of sightings from the surveys. As expected, the probability of a right whale sighting increased with increasing survey effort but leveled off when effort was >250 km^2^ (Figure 3A); this result supports our decision to model survey effort as a smoothed covariate rather than as an offset term. The SST for sampling units with a right whale present ranged from 9.0 to 22.6°C, with a mean (± SE) of 14.7°C (±0.04). The GAM predicted the highest probabilities between 12 and 16°C (Figure 3B). Right whales were more likely to be sighted close to shore, particularly within 25 km of the coast (Figure 3C), and at intermediate depths, between 10 and 25 m (Figure 3D). Generally, sightings occurred in cooler waters far from the 22°C isotherm, with only one sighting made south or east of this isotherm. However due to the proximity of the Gulf Stream to shore, especially in the southern portion of the study area, whales were limited by land to a maximum distance from the 22°C isotherm, and the response to this variable reached a plateau near this distance (Figure 3E). The interaction term between semimonth and northing was remarkably useful for capturing temporal shifts in distribution due to right whale migration in and out of the SEUS. Consistent with sighting data, the GAM predicted the highest probability of a whale sighting in January and February. Across all semimonthly periods, sighting rates were highest in intermediate northings of our study area, between latitudes of approximately 29.5 and 31.0°N. The interaction of these variables, however, demonstrated that whales occur in more northerly areas as they arrive at the beginning of the calving season in December, shift farther south in the middle of the season in January and February, and return north as they depart from the SEUS in March (Figure 3F). Survey year was also a significant predictor variable, as some years (*e.g.*, 2007/2008 and 2008/2009) had higher sighting rates than others (Figure 3G, Table S3).

### Abundance Model

For the positive abundance model, the selected best model included six predictor variables as significant (SST, distance to the shoreline, distance to the 22°C SST isotherm, bottom depth, interaction between semimonthly period and UTM northing, and survey year) and explained 12.2% of the deviance for the presence-only data (Table 2). Adding survey effort increased the GCV score and did not significantly decrease the explained deviance for modeling the number of whales (Table S1B, Table S2B), so this term was excluded from the model.

**Table 2:** Summary of stepwise selection procedure for positive abundance model of right whales in the southeastern US.

Model	% Deviance	GCV	mean ASPE
null	0	0.4534	8.284
s(SemiMonth:Northing)	6.5	0.4261	7.981
s(SemiMonth:Northing)+Year	10.2	0.4126	7.842
s(SemiMonth:Northing)+Year+s(SST)	11.2	0.4082	7.822
s(SemiMonth:Northing)+Year+s(SST)+s(DistTo22Iso)	11.7	0.4066	7.819
s(SemiMonth:Northing)+Year+s(SST)+s(DistTo22Iso)+s(DistToShore)	11.8	0.4065	7.820
s(SemiMonth:Northing)+Year+s(SST)+s(DistTo22Iso)+s(DistToShore)+s(Depth)	12.2	0.4054	7.807

Predictor variables and abbreviations same as in Table 1.

**Figure 4:**
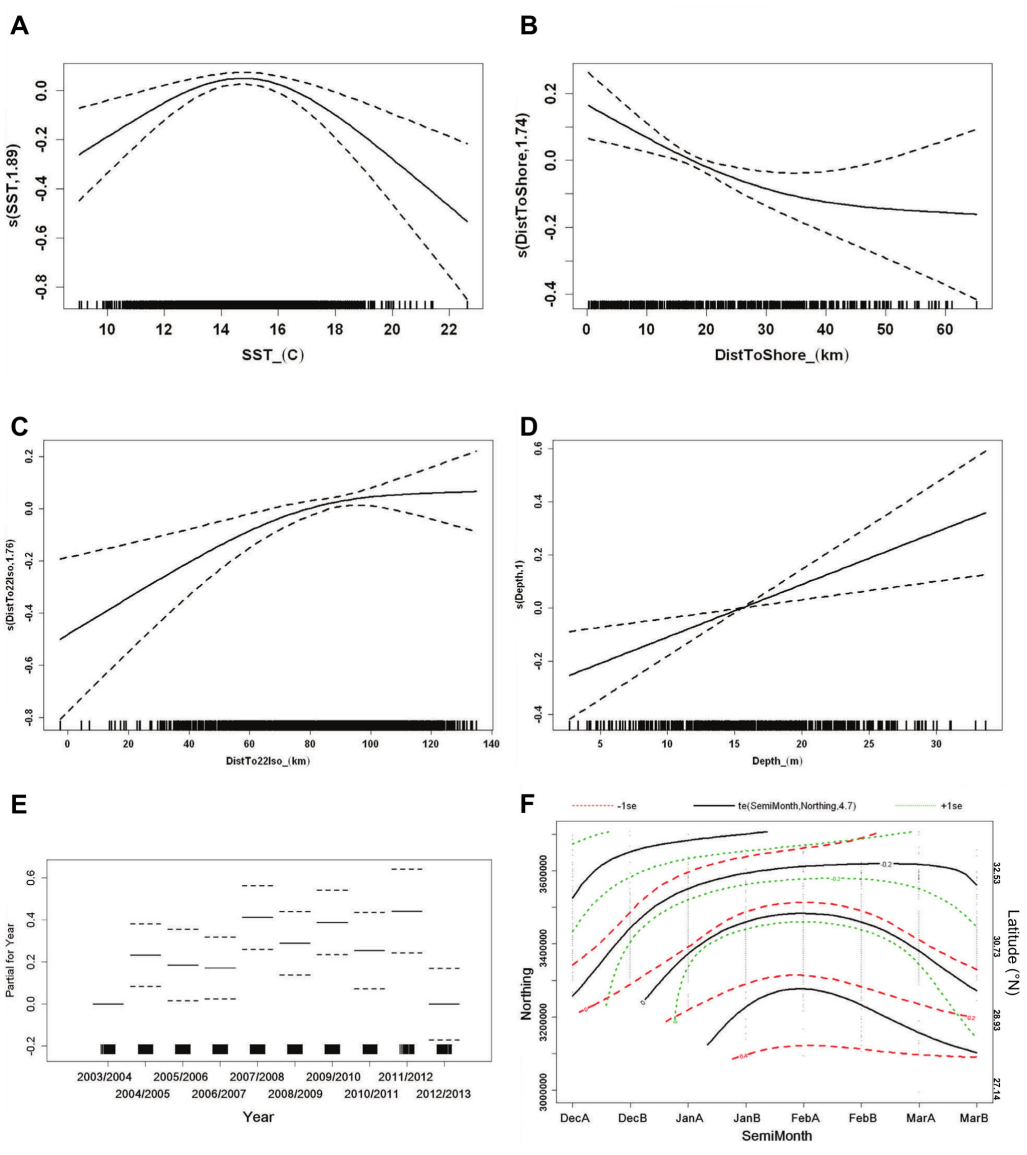
Smooth functions of predictor variables for positive abundance model of right whale sightings. Predictor variables and display same as in Figure 3.

The range of training data for the positive abundance model included only data from sampling units with whale sightings present, and the response for this model is the expected number of sighted whales per sampling unit, given whale presence. In general, smoothing functions in this model were similar to those in the presence-absence model, with more whales likely in sampling units with intermediate SST (Figure 4A), close to shore (Figure 4B), and in cooler waters far from the 22°C isotherm (Figure 4C). More whales were predicted, although with high uncertainty, near the upper limit of depth values with whales present (Figure 4D). Survey year was again significant, although the likelihood of multiple whales sighted in a sampling unit for a given year did not necessarily correspond with the overall sighting rate or probability of occurrence (Figure 4E, Table S3). Of the sampling units with whales present, multiple whales were more likely in sampling units at southerly northings and when whale densities were greatest, from late January through late February (Figure 4F). Smoothing functions for the positive abundance model generally had a higher standard error than those for the presence-absence model due in part to the smaller sample size.

### Model Predictions

The final, combined hurdle model was successful at predicting the number of observed whales, as deviance residuals were overall close to zero (mean ± SE = −0.219±0.003). However, examination of the model residuals revealed that, in general, the model tended to overpredict when whales were not sighted (*i.e.,* predicting whale occurrence in sampling units with no whales observed) and underpredict when whales were sighted. For sampling units where no whales were sighted, the mean observed number of whales – the mean predicted number of whales = 0–0.106. For sampling units where whales were sighted, the mean observed number of whales – the mean predicted number of whales = 3.027–0.524. Excluding the last semimonthly period from March 2013, we found no evidence for spatial autocorrelation of the residuals during any of the semimonthly periods, as all Moran’s *I* values approximated zero (mean = 0.030, range = −0.002 to 0.158). Moderate spatial autocorrelation was observed for late March 2013 (Moran’s *I* = 0.376), although no sightings were made during this semimonth and all residuals were thus negative.

**Figure 5:**
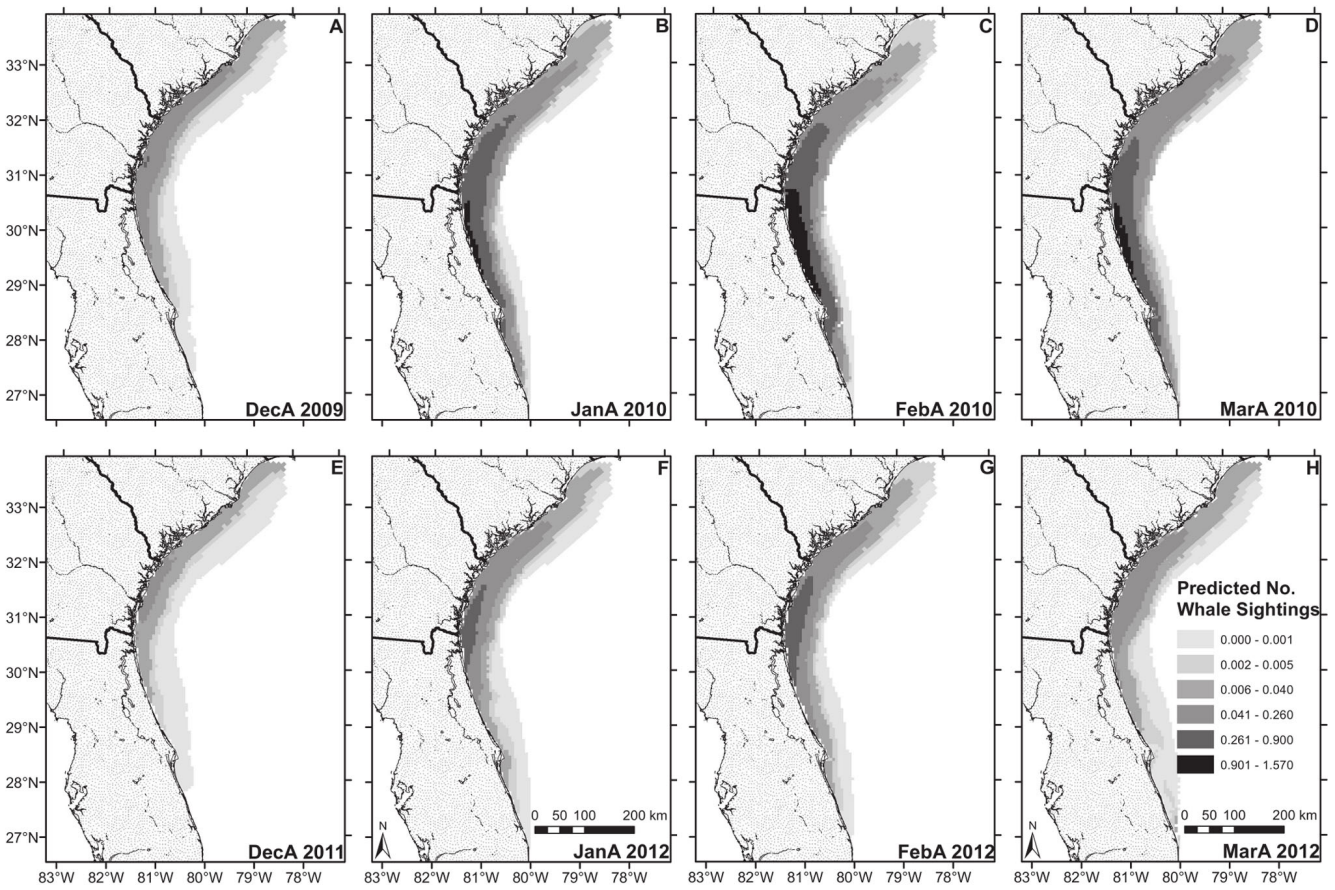
Predicted right whale relative abundance. Values represent predicted number of sighted right whales per grid cell (assuming uniform survey effort) during the 2009/2010 calving season (a relatively cold season with high sighting rates) for December 1–15 (A), January 1–15 (B), February 1–15 (C), and March 1–15 (D); and during the 2011/2012 calving season (a relatively warm season with low sighting rates) for December 1–15 (E), January 1–15 (F), February 1–15 (G), and March 1–15 (H).

Using the selected hurdle model, we created hindcasts predicting the spatial distribution of whales for all semimonthly periods in the study. With the predict.gam function in the mgcv package, the observed values for predictor variables (except survey effort) in all grid cells were used to generate the predicted and standard error estimates for probability of a whale sighting and number of whales sighted from the first and second steps of the hurdle model, respectively. Predicted relative abundance was then calculated by multiplying the probability of occurrence by the expected number of sighted whales. For these predictions, survey effort was set constant for all grid cells (at 250 km^2^, near which the smoothing function for the presence-absence model reached a plateau) to eliminate the confounding effect of variable survey effort on number of sightings. We did not extrapolate our results or predictions outside the range of our sampled data (*e.g.*, where depth was >70 m, the maximum value in our training data), nor did we make predictions for cells with missing data (*e.g.*, where SST data were lacking due to cloud interference). For illustrative purposes, we have included prediction maps for the first 15 days of each month for the 2009/2010 and 2011/2012 seasons (Figure 5). In general, sea surface temperatures were near average in December 2009 but colder than average for the remainder of the 2009/2010 season (Figure S1), and this season had intermediate sighting rates relative to other years in our data set (Figure 3G, Table S3). In contrast, the 2011/2012 season was warmer than average (Figure S1) and had low observed sighting rates relative to other years in our data set (Figure 3G, Table S3). Consistent with the smoothing functions and observed sightings from surveys, the greatest concentrations of predicted whale sightings for all semimonths occurred close to shore, in the relatively shallow and cooler waters west of the Gulf Stream (Figure 5). High predicted sighting rates were more common and distributed farther south in January and February than in December and March (Figure 5). Because 2009/2010 had a greater partial effect for the year term (Figure 3G), this season had higher predicted sighting rates overall compared to 2011/2012 (Figure 5). Notably, areas within the SEUS with the highest predicted number of whales were located farther south in a cold year (2009/2010; *e.g.*, Figure 5B) compared to a warm year (2011/2012; *e.g.*, Figure 5F). The presence-absence model seemed to drive the pattern for predicted relative abundance (Pearson correlation coefficient between predicted probability of presence and predicted relative abundance = 0.98); standard errors were generally larger for cells with high predicted probability of occurrence.

### Segregation of Calves

**Figure 6:**
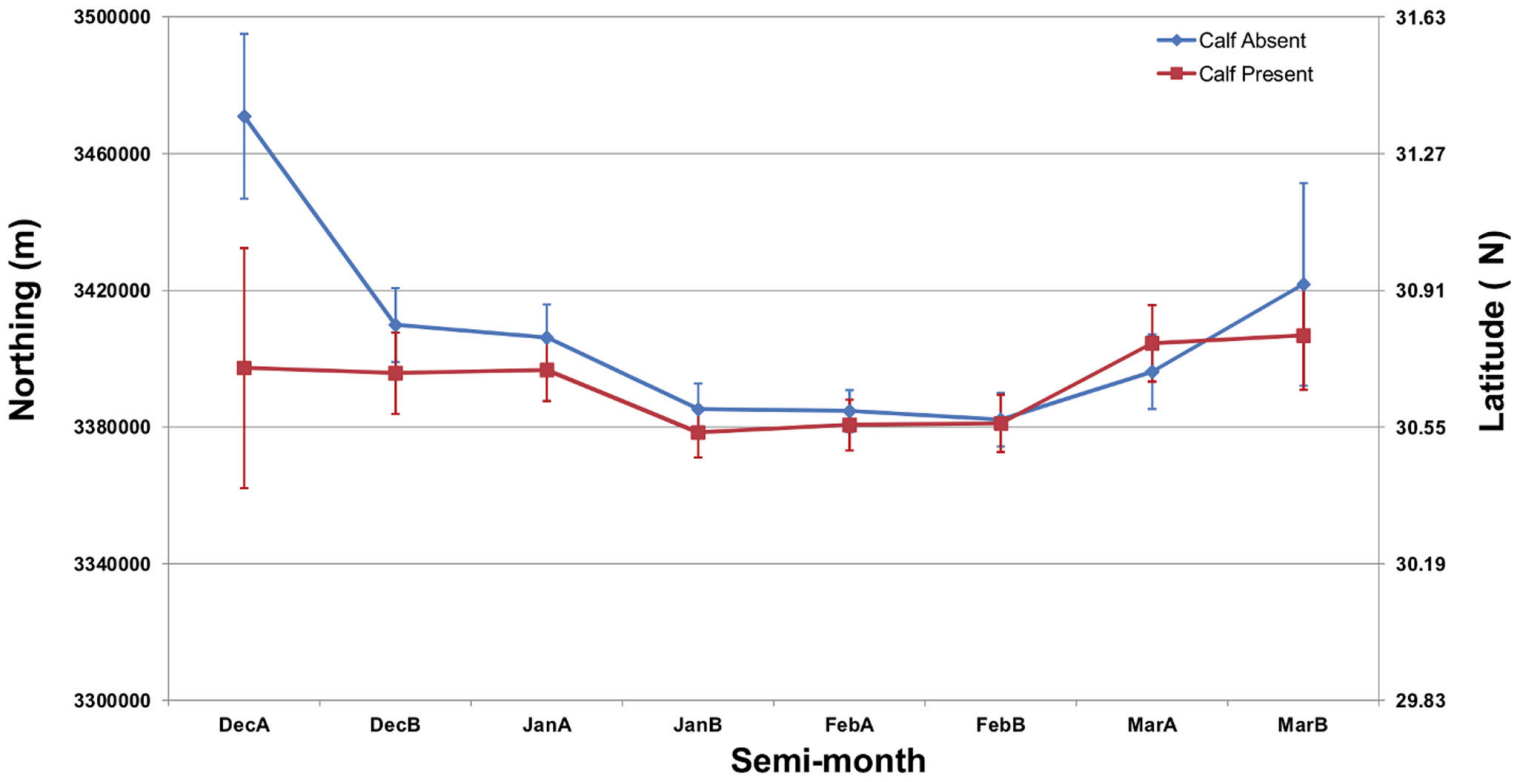
UTM northings for right whale sightings in the southeastern United States. Values indicate mean (±2 standard errors) UTM northing of sightings for each semimonthly period during all calving seasons. Sightings with a calf present displayed as red squares; sightings without a calf displayed as blue diamonds.

Of the total 3286 sightings, 1344 (41%) were of unaccompanied cow-calf pairs and 1919 (58%) were sightings without calves (*i.e.,* juveniles, adult males, non-calving adult females, pregnant females, and females that had lost a calf that year). The remaining 1% of sightings was composed of a calf by itself (6), two cow-calf pairs in close association (4), or a cow-calf pair accompanied by other individuals (13). We were able to extract SST data for 459 sightings with a calf present and 675 sightings with a calf absent, and all other environmental data for 1349 sightings with a calf present and 1907 sightings with a calf absent. Sightings with a calf present were made in locations significantly shallower and closer to shore and tended to occur in warmer water than sightings without calves, although the differences were small and there was extensive overlap in the range of values between group types (Table 3). Distance to the 22°C isotherm did not differ significantly among groups (Table 3). UTM northing did not differ significantly between groups with or without calves for any semimonthly period except the first period in December, when sightings without calves occurred farther north (*p*<0.01; Figure 6).

**Table 3:** Mean (range) environmental conditions at locations of right whale sightings with a calf present or absent.

Variable	Calf Present	Calf Absent	z-score	*p*
Depth (m)	14.9 (3.0–34.0)	15.5 (2.0–33.0)	−2.95	0.003
DistToShore (km)	17.6 (0.2–62.1)	18.5 (0.1–66.2)	−2.11	0.035
SST (°C)	14.9 (9.9–21.3)	14.7 (8.9–22.9)	−1.84	0.066
DistTo22Iso (km)	86.2 (11.7–135.3)	85.7 (−3.9–132.3)	−0.44	0.664

Environmental variables include bottom depth, distance to the shoreline (DistToShore), sea surface temperature (SST), and distance to the 22°C SST isotherm (DistTo22Iso).

## Discussion

In agreement with previous studies, our results indicate that GAMs are a useful tool for relating cetacean distribution to environmental variables and predicting cetacean occurrence and relative density based upon those variables [26], [36]. The development of a GAM, along with the ability to collect environmental data via remote sensing and to apply GIS processing techniques, permitted us to interpolate our results and estimate relative abundance of right whales, with associated standard error, in regions of our study area not sampled by aerial surveys. The flexibility of GAMs allowed right whale encounter rates to be modeled as a complex, nonlinear response to predictor variables, and a hurdle model approach allowed us to deal with zero-inflated data from surveys of a rare species summarized at a high spatial resolution. By using dynamic predictor variables and accounting for migration patterns, we were able to model changes in whale distribution through time.

Concordant with other studies in the SEUS [8], [9], [10], bottom depth and SST were significant predictors of right whale distribution; right whales were more likely to be sighted in waters 10 to 25 m deep and 12 to 16°C SST. Fonnesbeck *et al.* [9] and Keller *et al.* [10] also found year to be a significant factor variable related to differences in the total number of whales in the SEUS. Based on SST at sighting locations, Keller *et al.* [5], [10] suggested that right whales likely avoid the Gulf Stream, although they did not include a term to represent Gulf Stream variability in their model (such as distance to the 22°C SST isotherm). Distance to shore was a significant predictor in our model, in contrast to these previous studies which either did not consider this term [8], [9] or limited model complexity to four terms [10]. The range of preferred SST and avoidance of waters >22°C SST may be related to physiological optima and constraints on thermoregulation and growth, particularly in pregnant or nursing females and newborn calves [5], [37], [38]. It is less clear why whales were more common in shallow water close to shore, but it has been hypothesized that predator avoidance, weaker currents, and calm waters provided by these physical factors may be advantageous to calves and juveniles that are still developing swimming skills or more susceptible to predation [8], [34]. Unfortunately, it will be difficult to adequately address these hypotheses unless predator abundance and wave/surface roughness data become available at finer temporal and spatial resolutions.

Our model also included the interaction between northing and semimonthly period to account for whale migration during the calving season. Both Good [8] and Keller *et al.* [10] predicted suitable habitat off northern Georgia and South Carolina throughout the winter. We, however, predict that these areas have relatively lower encounter rates compared to core-use areas off northern Florida and southern Georgia, particularly during the middle of the winter (Figure 5), congruent with sighting data (Figure 2). We believe that this difference in predictions is driven by the additional data with low sighting rates from the SC-GA surveys and by the inclusion of the latitude/semimonth interaction term in our model, which accounted for latitudinal shifts in whale distribution that were independent of SST variability. This is the first habitat model, to our knowledge, that explicitly considers a migration process in an area outside of the species’ feeding grounds. Although the latitude/semimonth interaction term is only a proxy for the inherent processes that truly drive the migration timing, and it limits the application of the model to the area analyzed, it nevertheless improved the predictive capability of our model and is therefore useful for identifying areas in the SEUS with high whale encounter rates and predicting how these encounter rates change throughout a winter season. Moreover, the ecological factors that may influence the timing of whale migration into and within the SEUS (e.g., predation risk, calm seas, and historical distribution of con-specifics) are difficult to quantify and remain unknown. Although our model predicts that sightings are less common and whales likely spend less time in the area off northern Georgia and South Carolina, this does not mean that right whales do not use (and possibly give birth in) these areas. We hypothesize that, in most years, these more northerly areas are part of the right whale migration corridor [39], [40]. Whales may display behaviors in these corridors that affect detectability (if they spend more time below the surface) and may have shorter residence times there than in the core wintering area. We therefore suggest that a separate model be created to characterize the right whale migration corridor when more data on migrating whales become available. Variability in sea surface roughness (not addressed in this study) and the occurrence of Gulf Stream meanders north of Charleston, SC, which generate onshore movements of unsuitably warm water [19], [41], may yield a less stable habitat for whales in the northern areas of the SEUS. Nevertheless, we agree with Good [8] and Keller *et al.* [10] that in some years the core wintering area extends farther north than the current critical habitat boundary (*e.g.*, Figure 5G). Although extrapolating model results can help identify potential areas for future surveys [10], [12], the different predictions among studies here highlight the importance of validating and updating models with additional data and may emphasize that caution is needed when applying model results beyond the spatial and temporal extent of training data [42].

Environmental variability among years leads to differences in both the total number of whales that migrate to the SEUS and the spatial distribution of whales once they arrive there (Figure 5, Keller *et al.* [10]). Variability in right whale spatial distribution among years seems to be driven by local conditions such as water temperature and location of the Gulf Stream, which vary in the SEUS among years and within a calving season (Figure S1). For example, whales are distributed farther south in relatively cold years (Figure 5), presumably to occupy areas of preferred SST. However, the number of whales that migrate to the SEUS in a given year and the amount of time they spend there are likely influenced by factors outside the SEUS [43], [44]. Greene *et al.* [43] and Kenney [44] suggest that female right whale calving rates (and consequently the number of calving females that migrate to the SEUS each year) are determined by the number of females available to calve and by recovery times for reproductive females, ultimately influenced by food availability in summer foraging grounds. Encounter rates of whales will also be affected by the duration of whale residence in the SEUS, which may be a consequence of demographics (with calving females possibly having longer residence times than other whales [45]), energy reserves, changes in weather, or other, unknown factors. Although including year as a factor variable significantly improved our model fit and the ability to hindcast whale relative abundance for the years analyzed in our study, it precluded the ability to predict the magnitude of whale densities for years beyond our data set. Replacement of this term with a variable that describes the number and demography of right whales predicted to migrate to the wintering grounds each year (*e.g.*, [43]) would greatly improve the predictive capabilities of our model, particularly for forecasting the true value of whale encounter rates. Overall, the results of this study demonstrate that whale abundance and distribution vary within and between years. Recognizing and understanding this temporal variability can better inform the location and timing of management actions as whale distribution changes throughout a season, which would result in more effective risk mitigation and population monitoring of right whales.

While the final model explained the most variation in our dataset, overdispersion (partly caused by the high spatio-temporal resolution of our sampling units, the rarity of right whale sightings, and the potentially high number of false absences generated by aerial survey data) created challenges to achieving a good model fit. Indeed, it is common for GAMs modeling cetacean occurrence at a high spatial resolution, and non-normal models in general, to explain only a small proportion of the deviance in a dataset [26], [36], [46]. For example, our model frequently under-estimated the number of whales in grid cells with observed sightings, and this may be related to how the data were aggregated. Rather than predicting a high sighting probability in a single grid cell, the model smoothed predicted whale occurrence over several nearby cells with similar, preferred habitat conditions; in this regard, the model predictions may describe more accurately where a whale may be located over the span of two weeks (*i.e.,* temporarily occupying multiple nearby cells) than would an aerial survey sighting fixed in time and location. Conversely, the model tended to overpredict whale occurrence in areas where no whales were sighted. The small population size of right whales could have resulted in the absence of whales from suitable habitat [47]. Additionally, the lack of a sighting does not necessarily mean that whales were truly absent. Whales might not have been detected due to availability bias (*e.g.*, whales were submerged [48]), perception bias (*e.g.*, observer error [49]), or incomplete temporal coverage by surveys (*i.e.,* not flying at night, not flying during poor weather). In consideration of these limitations of aerial surveys and the confounding effect of unequal survey effort on the number of sightings, we believe that predictive habitat models can better characterize the distribution of whales than can sightings-per-unit-effort data (*e.g.*, Figure 2C). Rather than relying solely on costly surveys with imperfect detection probabilities and limited coverage, predictions from these models can be particularly helpful in making management decisions that require risk assessment based on expected whale distribution [50].

Whale groups with a calf present were sighted in waters that were shallower and closer to shore than were other whale groups. Even though shallow, nearshore locations in our study area generally had the coldest water, there was also a trend for groups with calves to be sighted in slightly warmer water. Sightings with a calf present occurred farther south during early December, although this finding is confounded by few sightings of calves early in the calving season and the fact that calves-of-the-year are not likely to be migrating from the north at this time. Studies of baleen whales on their wintering grounds have also found cow-calf pairs closer to shore and in shallower water than other demographic groups [32], [33], [51]. Differences in habitat preference may be a result of different energetic constraints or predation risks between demographic groups. Although our results were statistically significant and supported by other studies, the differences between groups were minimal, the range of values for environmental variables were very similar, and sightings of groups with calves compared with those without calves were not spatially segregated at the scale and spatial extent of our model. We therefore conclude that separate habitat models for cow-calf pairs and other demographic groups in the SEUS are not warranted and are impracticable at the scale used in this study, and management areas for one group will likely be equally effective for the other. Despite the large number of sightings in our data, however, it was extremely rare for a cow-calf pair to be sighted with another whale. Thus, at a fine scale, it appears that cow-calf pairs are segregated from other whales, but this pattern may be influenced more by social dynamics (*e.g.*, harassment avoidance; [34], [52]) than by environmental features.

Our analysis presents an example of the use of static and dynamic environmental variables to predict species distribution and characterize habitat preferences of a migratory species in a dynamic seascape. Using temporally dynamic variables, such as SST, isotherms, and a seasonal migration index, we were able to predict semimonthly right whale distribution. Although our framework does not estimate absolute densities, it provides additional insights into how whale distribution changes over time compared to a single depiction of instantaneous density. This framework could be applied to other highly mobile migratory species in variable habitats that may benefit from dynamic management actions [6], [53]. The model results corroborate that right whale distribution varies within and among years. We believe that the addition of data from a broader survey area relative to previous studies, and accounting for migration patterns, improved the model by predicting how whale distribution changes within a calving season and by separating core-use areas from short-term-use areas in the migration corridor. Hindcast predictions from this model can be used to assess risks to whales and effectiveness of management actions. Improvements of the model, including the addition of parameters for predicting the total number of whales that migrate to the wintering grounds [43], would allow for near-real-time forecasts of whale distribution that could be used to better inform management decisions. We encourage future evaluations of this and other habitat models as additional data, particularly from other predictors and from surveys in areas previously receiving little coverage, become available.

## Supporting Information

Figure S1 **Average daily sea surface temperatures for December through March.** Data obtained from the National Data Buoy Center (http://www.ndbc.noaa.gov) at Gray’s Reef (top panel, station 41008, 31.400°N 80.868°W) and at St. Augustine (bottom panel, station SAUF1, 29.857°N 81.265°W). Data include 2009/2010 (blue line), 2011/2012 (red line), long-term historical average with 95% confidence intervals (1991/1992 and 1997/1998–2012/2013 at Gray’s Reef; 1988/1989–2001/2002 and 2004/2005–2011/2012 at St. Augustine), and long-term historical range.
(DOCX)

Table S1 A. Summary of all models tested in stepwise selection procedure for presence-absence models of right whales in the southeastern United States. Predictor variables include interaction between semimonthly period and UTM northing, distance to the shoreline (DistToShore), survey year, survey effort, sea surface temperature (SST), bottom depth, distance to the 22°C SST isotherm (DistTo22Iso), and slope. Smoothed covariates indentified by “s()”. Evaluation criteria include the proportion of deviance explained, generalized cross validation score (GCV), and mean average squared prediction error (ASPE) from a five-fold cross-validation. The best model at each step is in bold. B. Summary of all models tested in stepwise selection procedure for positive abundance models of right whales in the southeastern United States. Predictor variables and abbreviations same as in Table S1A. The best model at each step is in bold.
(DOCX)

Table S2 A. Results of analysis of deviance tests comparing presence-absence models at each step of the stepwise selection procedure. Models at each step refer to the respective best model (in bold) from Table S1A. Reductions in deviance, *F*-statistics, and *p*-values compare each model to the model in the previous step. B. Results of analysis of deviance tests comparing positive abundance models at each step of the stepwise selection procedure. Models at each step refer to the respective best model (in bold) from Table S1B. Reductions in deviance, *F*-statistics, and *p*-values compare each model to the model in the previous step.
(DOCX)

Table S3 **Number of observed sightings, number of observed whales (not unique individuals), total survey effort, and overall sighting rate (sightings/1000 km^2^ surveyed) for each calving season from our data set.**
(DOCX)
