# Supplementary material for: Wintering Habitat Model for the North Atlantic Right Whale (Eubalaena glacialis) in the Southeastern United States
Source: PLoS One. 2014 Apr 16;9(4):e95126. doi: 10.1371/journal.pone.0095126 (PMC3989274; doi:10.1371/journal.pone.0095126)
Supplement: Figure S1 — Average daily sea surface temperatures for December through March. Data obtained from the National Data Buoy Center (http://www.ndbc.noaa.gov) at Gray’s Reef (top panel, station 41008, 31.400°N 80.868°W) and at St. Augustine (bottom panel, station SAUF1, 29.857°N 81.265°W). Data include 2009/2010 (blue line), 2011/2012 (red line), long-term historical average with 95% confidence intervals (1991/1992 and 1997/1998–2012/2013 at Gray’s Reef; 1988/1989–2001/2002 and 2004/2005–2011/2012 at St. Augustine), and long-term historical range. (DOCX) [file pone.0095126.s001.docx]

Figure S1. Average daily sea surface temperatures for December through March. Data obtained from the National Data Buoy Center (<http://www.ndbc.noaa.gov>) at Gray’s Reef (top panel, station 41008, 31.400°N 80.868°W) and at St. Augustine (bottom panel, station SAUF1, 29.857°N 81.265°W). Data include 2009/2010 (blue line), 2011/2012 (red line), long-term historical average with 95% confidence intervals (1991/1992 and 1997/1998–2012/2013 at Gray’s Reef; 1988/1989–2001/2002 and 2004/2005–2011/2012 at St. Augustine), and long-term historical range.

°
