# Supplementary material for: Wintering Habitat Model for the North Atlantic Right Whale (Eubalaena glacialis) in the Southeastern United States
Source: PLoS One. 2014 Apr 16;9(4):e95126. doi: 10.1371/journal.pone.0095126 (PMC3989274; doi:10.1371/journal.pone.0095126)
Supplement: Table S1 — A. Summary of all models tested in stepwise selection procedure for presence-absence models of right whales in the southeastern United States. Predictor variables include interaction between semimonthly period and UTM northing, distance to the shoreline (DistToShore), survey year, survey effort, sea surface temperature (SST), bottom depth, distance to the 22°C SST isotherm (DistTo22Iso), and slope. Smoothed covariates indentified by “s()”. Evaluation criteria include the proportion of deviance explained, generalized cross validation score (GCV), and mean average squared prediction error (ASPE) from a five-fold cross-validation. The best model at each step is in bold. B. Summary of all models tested in stepwise selection procedure for positive abundance models of right whales in the southeastern United States. Predictor variables and abbreviations same as in Table S1A. The best model at each step is in bold. (DOCX) [file pone.0095126.s002.docx]

Table S1A. Summary of all models tested in stepwise selection procedure for presence-absence models of right whales in the southeastern United States. Predictor variables include interaction between semimonthly period and UTM northing, distance to the shoreline (DistToShore), survey year, survey effort, sea surface temperature (SST), bottom depth, distance to the 22°C SST isotherm (DistTo22Iso), and slope. Smoothed covariates indentified by “s()”. Evaluation criteria include the proportion of deviance explained, generalized cross validation score (GCV), and mean average squared prediction error (ASPE) from a five-fold cross-validation. The best model at each step is in bold.

| Step | Model | % Deviance | GCV | mean ASPE |
| --- | --- | --- | --- | --- |
| **1** | **null** | **0.0** | **0.3417** | **0.0392** |
|  |  |  |  |  |
| 2 | s(Effort) | 9.1 | 0.3108 | 0.0378 |
| 2 | s(Depth) | 4.6 | 0.3260 | 0.0386 |
| **2** | **s(SemiMonth:Northing)** | **10.3** | **0.3065** | **0.0377** |
| 2 | s(DistToShore) | 4.8 | 0.3253 | 0.0386 |
| 2 | s(SST) | 3.6 | 0.3293 | 0.0388 |
| 2 | Year | 2.6 | 0.3328 | 0.0388 |
| 2 | s(DistTo22Iso) | 4.6 | 0.3261 | 0.0387 |
| 2 | s(Slope) | 0.1 | 0.3415 | 0.0392 |
|  |  |  |  |  |
| 3 | s(SemiMonth:Northing) + s(Effort) | 13.8 | 0.2945 | 0.0368 |
| 3 | s(SemiMonth:Northing) + s(Depth) | 15.5 | 0.2889 | 0.0364 |
| **3** | **s(SemiMonth:Northing) + s(DistToShore)** | **15.7** | **0.2882** | **0.0364** |
| 3 | s(SemiMonth:Northing) + s(SST) | 13.6 | 0.2953 | 0.0371 |
| 3 | s(SemiMonth:Northing) + Year | 13.3 | 0.2964 | 0.0369 |
| 3 | s(SemiMonth:Northing) + s(DistTo22Iso) | 14.9 | 0.2909 | 0.0367 |
| 3 | s(SemiMonth:Northing) + s(Slope) | 10.4 | 0.3061 | 0.0377 |
|  |  |  |  |  |
| 4 | s(SemiMonth:Northing) + s(DistToShore) + s(Effort) | 17.5 | 0.2821 | 0.0359 |
| 4 | s(SemiMonth:Northing) + s(DistToShore) + s(Depth) | 16.6 | 0.2853 | 0.0361 |
| 4 | s(SemiMonth:Northing) + s(DistToShore) + s(SST) | 17.0 | 0.2839 | 0.0361 |
| **4** | **s(SemiMonth:Northing) + s(DistToShore) + Year** | **18.8** | **0.2777** | **0.0352** |
| 4 | s(SemiMonth:Northing) + s(DistToShore) + s(DistTo22Iso) | 17.0 | 0.2836 | 0.0362 |
| 4 | s(SemiMonth:Northing) + s(DistToShore) + s(Slope) | 15.8 | 0.2878 | 0.0364 |
|  |  |  |  |  |
| **5** | **s(SemiMonth:Northing) + s(DistToShore) + Year + s(Effort)** | **20.9** | **0.2706** | **0.0345** |
| 5 | s(SemiMonth:Northing) + s(DistToShore) + Year + s(Depth) | 19.6 | 0.2748 | 0.0348 |
| 5 | s(SemiMonth:Northing) + s(DistToShore) + Year + s(SST) | 20.0 | 0.2734 | 0.0348 |
| 5 | s(SemiMonth:Northing) + s(DistToShore) + Year + s(DistTo22Iso) | 19.7 | 0.2745 | 0.0350 |
| 5 | s(SemiMonth:Northing) + s(DistToShore) + Year + s(Slope) | 18.9 | 0.2773 | 0.0351 |
|  |  |  |  |  |
| 6 | s(SemiMonth:Northing) + s(DistToShore) + Year + s(Effort) + s(Depth) | 21.6 | 0.2681 | 0.0342 |
| **6** | **s(SemiMonth:Northing) + s(DistToShore) + Year + s(Effort) + s(SST)** | **22.3** | **0.2658** | **0.0342** |
| 6 | s(SemiMonth:Northing) + s(DistToShore) + Year + s(Effort) + s(DistTo22Iso) | 21.8 | 0.2674 | 0.0343 |
| 6 | s(SemiMonth:Northing) + s(DistToShore) + Year + s(Effort) + s(Slope) | 21.0 | 0.2700 | 0.0344 |
|  |  |  |  |  |
| **7** | **s(SemiMonth:Northing) + s(DistToShore) + Year + s(Effort) + s(SST) + s(Depth)** | **22.6** | **0.2647** | **0.0340** |
| 7 | s(SemiMonth:Northing) + s(DistToShore) + Year + s(Effort) + s(SST) + s(DistTo22Iso) | 22.5 | 0.2650 | 0.0341 |
| 7 | s(SemiMonth:Northing) + s(DistToShore) + Year + s(Effort) + s(SST) + s(Slope) | 22.4 | 0.2653 | 0.0341 |
|  |  |  |  |  |
| **8** | **s(SemiMonth:Northing) + s(DistToShore) + Year + s(Effort) + s(SST) + s(Depth) + s(DistTo22Iso)** | **22.8** | **0.2642** | **0.0340** |
| 8 | s(SemiMonth:Northing) + s(DistToShore) + Year + s(Effort) + s(SST) + s(Depth) + s(Slope) | 22.7 | 0.2643 | 0.0340 |

Table S1B. Summary of all models tested in stepwise selection procedure for positive abundance models of right whales in the southeastern United States. Predictor variables and abbreviations same as in Table S1A. The best model at each step is in bold.

| Step | Model | % Deviance | GCV | mean ASPE |
| --- | --- | --- | --- | --- |
| **1** | **null** | **0** | **0.4534** | **8.284** |
|  |  |  |  |  |
| 2 | s(Effort) | 0.9 | 0.4502 | 8.244 |
| 2 | s(Depth) | 0.6 | 0.4514 | 8.246 |
| **2** | **s(SemiMonth:Northing)** | **6.5** | **0.4261** | **7.981** |
| 2 | s(DistToShore) | 0.4 | 0.4522 | 8.286 |
| 2 | s(SST) | 1.4 | 0.4481 | 8.269 |
| 2 | Year | 4.1 | 0.4384 | 8.143 |
| 2 | s(DistTo22Iso) | 2.1 | 0.4447 | 8.189 |
| 2 | s(Slope) | 0.3 | 0.4526 | 8.308 |
|  |  |  |  |  |
| 3 | s(SemiMonth:Northing) + s(Effort) | 6.7 | 0.4257 | 7.967 |
| 3 | s(SemiMonth:Northing) + s(Depth) | 6.5 | 0.4265 | 7.985 |
| 3 | s(SemiMonth:Northing) + s(DistToShore) | 6.7 | 0.4258 | 7.979 |
| 3 | s(SemiMonth:Northing) + s(SST) | 7.1 | 0.4237 | 8.009 |
| **3** | **s(SemiMonth:Northing) + Year** | **10.2** | **0.4126** | **7.842** |
| 3 | s(SemiMonth:Northing) + s(DistTo22Iso) | 8.4 | 0.4183 | 7.874 |
| 3 | s(SemiMonth:Northing) + s(Slope) | 6.6 | 0.4261 | 7.998 |
|  |  |  |  |  |
| 4 | s(SemiMonth:Northing) + Year + s(Effort) | 10.2 | 0.4128 | 7.845 |
| 4 | s(SemiMonth:Northing) + Year + s(Depth) | 10.2 | 0.4127 | 7.847 |
| 4 | s(SemiMonth:Northing) + Year + s(DistToShore) | 10.6 | 0.4114 | 7.825 |
| **4** | **s(SemiMonth:Northing) + Year + s(SST)** | **11.2** | **0.4082** | **7.822** |
| 4 | s(SemiMonth:Northing) + Year + s(DistTo22Iso) | 11.2 | 0.4083 | 7.798 |
| 4 | s(SemiMonth:Northing) + Year + s(Slope) | 10.4 | 0.4124 | 7.855 |
|  |  |  |  |  |
| 5 | s(SemiMonth:Northing) + Year + s(SST) + s(Effort) | 11.3 | 0.4084 | 7.822 |
| 5 | s(SemiMonth:Northing) + Year + s(SST) + s(Depth) | 11.2 | 0.4085 | 7.826 |
| 5 | s(SemiMonth:Northing) + Year + s(SST) + s(DistToShore) | 11.5 | 0.4076 | 7.814 |
| **5** | **s(SemiMonth:Northing) + Year + s(SST) + s(DistTo22Iso)** | **11.7** | **0.4066** | **7.819** |
| 5 | s(SemiMonth:Northing) + Year + s(SST) + s(Slope) | 11.4 | 0.4081 | 7.833 |
|  |  |  |  |  |
| 6 | s(SemiMonth:Northing) + Year + s(SST) + s(DistTo22Iso) + s(Effort) | 11.8 | 0.4069 | 7.820 |
| 6 | s(SemiMonth:Northing) + Year + s(SST) + s(DistTo22Iso) + s(Depth) | 11.8 | 0.4066 | 7.822 |
| **6** | **s(SemiMonth:Northing) + Year + s(SST) + s(DistTo22Iso) + s(DistToShore)** | **11.8** | **0.4065** | **7.820** |
| 6 | s(SemiMonth:Northing) + Year + s(SST) + s(DistTo22Iso) + s(Slope) | 11.8 | 0.4066 | 7.835 |
|  |  |  |  |  |
| 7 | s(SemiMonth:Northing) + Year + s(SST) + s(DistTo22Iso) + s(DistToShore) + s(Effort) | 11.9 | 0.4067 | 7.821 |
| **7** | **s(SemiMonth:Northing) + Year + s(SST) + s(DistTo22Iso) + s(DistToShore) + s(Depth)** | **12.2** | **0.4054** | **7.807** |
| 7 | s(SemiMonth:Northing) + Year + s(SST) + s(DistTo22Iso) + s(DistToShore) + s(Slope) | 11.9 | 0.4067 | 7.836 |
|  |  |  |  |  |
| 8 | s(SemiMonth:Northing) + Year + s(SST) + s(DistTo22Iso) + s(DistToShore) + s(Depth) + s(Effort) | 12.2 | 0.4055 | 7.807 |
| 8 | s(SemiMonth:Northing) + Year + s(SST) + s(DistTo22Iso) + s(DistToShore) + s(Depth) + s(Slope) | 12.2 | 0.4056 | 7.819 |
