# Supplementary material for: Wintering Habitat Model for the North Atlantic Right Whale (Eubalaena glacialis) in the Southeastern United States
Source: PLoS One. 2014 Apr 16;9(4):e95126. doi: 10.1371/journal.pone.0095126 (PMC3989274; doi:10.1371/journal.pone.0095126)
Supplement: Table S2 — A. Results of analysis of deviance tests comparing presence-absence models at each step of the stepwise selection procedure. Models at each step refer to the respective best model (in bold) from Table S1A. Reductions in deviance, F-statistics, and p-values compare each model to the model in the previous step. B. Results of analysis of deviance tests comparing positive abundance models at each step of the stepwise selection procedure. Models at each step refer to the respective best model (in bold) from Table S1B. Reductions in deviance, F-statistics, and p-values compare each model to the model in the previous step. (DOCX) [file pone.0095126.s003.docx]

Table S2A. Results of analysis of deviance tests comparing presence-absence models at each step of the stepwise selection procedure. Models at each step refer to the respective best model (in bold) from Table S1A. Reductions in deviance, *F*-statistics, and *p*-values compare each model to the model in the previous step.

| Model | Estimated df | Residual Deviance | Reduction in Deviance | *F* | *p* |
| --- | --- | --- | --- | --- | --- |
| null | 1.00 | 19183 |  |  |  |
| Step2 | 8.79 | 17202 | 1980.7 | 829.2 | <0.001 |
| Step3 | 10.89 | 16173 | 1029.3 | 1701.0 | <0.001 |
| Step4 | 19.89 | 15581 | 591.7 | 237.0 | <0.001 |
| Step5 | 21.87 | 15183 | 398.1 | 741.5 | <0.001 |
| Step6 | 23.55 | 14912 | 270.7 | 608.9 | <0.001 |
| Step7 | 25.45 | 14845 | 67.4 | 133.7 | <0.001 |
| Step8 | 27.44 | 14816 | 28.4 | 54.2 | <0.001 |

Table S2B. Results of analysis of deviance tests comparing positive abundance models at each step of the stepwise selection procedure. Models at each step refer to the respective best model (in bold) from Table S1B. Reductions in deviance, *F*-statistics, and *p*-values compare each model to the model in the previous step.

| Model | Estimated df | Residual Deviance | Reduction in Deviance | *F* | *p* |
| --- | --- | --- | --- | --- | --- |
| null | 1.00 | 1040.6 |  |  |  |
| Step2 | 6.59 | 973.2 | 67.4 | 28.4 | <0.001 |
| Step3 | 15.88 | 934.6 | 38.6 | 10.1 | <0.001 |
| Step4 | 16.69 | 924.0 | 10.7 | 32.7 | <0.001 |
| Step5 | 18.99 | 918.5 | 5.5 | 5.9 | 0.002 |
| Step6 | 20.16 | 917.3 | 1.2 | 2.4 | 0.114 |
| Step7 | 21.10 | 914.1 | 3.2 | 8.5 | 0.004 |
| Step8 | 22.71 | 913.2 | 0.9 | 1.4 | 0.248 |
