# Supplementary material for: Wintering Habitat Model for the North Atlantic Right Whale (Eubalaena glacialis) in the Southeastern United States
Source: PLoS One. 2014 Apr 16;9(4):e95126. doi: 10.1371/journal.pone.0095126 (PMC3989274; doi:10.1371/journal.pone.0095126)
Supplement: Table S3 — Number of observed sightings, number of observed whales (not unique individuals), total survey effort, and overall sighting rate (sightings/1000 km2 surveyed) for each calving season from our data set. (DOCX) [file pone.0095126.s004.docx]

Table S3. Number of observed sightings, number of observed whales (not unique individuals), total survey effort, and overall sighting rate (sightings/1000 km^2^ surveyed) for each calving season from our data set.

| Year | Observed Sightings | Observed Whales | Survey Effort (1000 km^2^) | Sighting Rate |
| --- | --- | --- | --- | --- |
| 2003/2004 | 137 | 259 | 312 | 0.44 |
| 2004/2005 | 305 | 732 | 418 | 0.73 |
| 2005/2006 | 252 | 581 | 448 | 0.56 |
| 2006/2007 | 306 | 669 | 387 | 0.79 |
| 2007/2008 | 516 | 1149 | 414 | 1.25 |
| 2008/2009 | 712 | 1542 | 407 | 1.75 |
| 2009/2010 | 435 | 1011 | 405 | 1.07 |
| 2010/2011 | 194 | 452 | 359 | 0.54 |
| 2011/2012 | 109 | 289 | 477 | 0.23 |
| 2012/2013 | 138 | 269 | 378 | 0.37 |
